# AffMB: affinity maturation analysis with SHM-guided B-cell lineage trees

**DOI:** 10.1093/bioinformatics/btaf346

**Published:** 2025-07-17

**Authors:** Jiaqi Luo, Yiping Zou, Shuai Cheng Li

**Affiliations:** Department of Computer Science, City University of Hong Kong, Kowloon Tong, Hong Kong, China; City University of Hong Kong Shenzhen Research Institute, Nanshan District, Shenzhen, 518000, China; Department of Computer Science, City University of Hong Kong, Kowloon Tong, Hong Kong, China; City University of Hong Kong Shenzhen Research Institute, Nanshan District, Shenzhen, 518000, China; Department of Computer Science, City University of Hong Kong, Kowloon Tong, Hong Kong, China; City University of Hong Kong Shenzhen Research Institute, Nanshan District, Shenzhen, 518000, China

## Abstract

**Motivation:**

B-cell lineage trees describe the evolutionary process of immunoglobulin genes during affinity maturation. Existing methods for building B-cell lineage trees generally do not guarantee the parent-to-child inheritance and accumulation of advantageous mutations under successive rounds of somatic hypermutation (SHM) and selection, and are often incompatible with repertoire input.

**Results:**

To address previous limitations, we developed AffMB (Affinity Maturation of B-cell receptor), a comprehensive toolkit for tracking affinity maturation through the generation and visualization of SHM-ordered, inheritance-based B-cell lineage trees from single-cell or bulk B-cell receptor sequencing data. The SHM-ordered inheritance tree algorithm outperformed state-of-the-art benchmarks in simulations. When applied to single-cell data from BNT162b2 vaccination (*n* = 42), AffMB demonstrated the ability to infer immunization responses and showed the feasibility of identifying potential high-affinity antibody sequences.

**Availability and implementation:**

AffMB is an open-source Python package that supports contig FASTA or AIRR rearrangement TSV inputs. The source code for AffMB is freely available at https://github.com/deepomicslab/AffMB.

## 1 Introduction

Affinity maturation of B cells is an iterative mutation-selection-expansion process that ultimately produces memory and effector B cells with high-affinity B-cell receptors (BCRs), either membrane-bound or secreted as antibodies. The hallmark of affinity maturation is somatic hypermutation (SHM) under extreme selection, where advantageous mutations predominantly occur in complementarity-determining regions (CDRs) that directly mediate antigen binding. B-cell phylogenetic trees can be reconstructed from BCR sequences to trace SHM-driven immunoglobulin (Ig) gene evolution during affinity maturation. However, unlike the general evolution of species over long timescales (e.g. millions of years), affinity maturation operates within days under extreme selective pressure. This unique biology allows coexistence of distant ancestors and recent progeny, resulting in sampled internal nodes in B-cell phylogenetic trees, a feature incompatible with conventional binary phylogenetic trees that assume all sampled nodes are tips (Barak *et al.* 2008, DeWitt *et al.* 2018, Yang *et al.* 2020, Abdollahi *et al.* 2023, Hoehn and Kleinstein, 2024). Ig/B-cell lineage trees (Barak *et al.* 2008, DeWitt *et al.* 2018, Yang *et al.* 2020, Abdollahi *et al.* 2023) were developed specifically for B-cell phylogenetics to address this disparity. Ig/B-cell lineage tree construction algorithms have evolved from maximum parsimony (Barak *et al.* 2008, DeWitt *et al.* 2018) to minimum spanning trees (MSTs) (Yang *et al.* 2020, Abdollahi *et al.* 2023), achieving greater computational efficiency while incorporating sequence abundance data to reflect clonal expansion dynamics.

Unlike conventional evolution, which spans long enough for environmental shifts that may favour backward evolution (i.e. reversed mutations), affinity maturation occurs rapidly under constant selective pressure. Selection is dictated by binding affinity to a fixed antigen, and the survival competition intensifies over generations. Consequently, reversed mutations are considered not likely to occur. Given the consistent mutation rate of 1–2 CDR mutations per generation (Teng and Papavasiliou 2007), neutral mutations become disadvantageous by failing to enhance affinity. Only beneficial mutations adding to affinity are likely to persist across generations. Since sampled BCR sequences represent survival through selection, we consider all observed CDR mutations advantageous and should thus be inherited.

**Figure 1.**
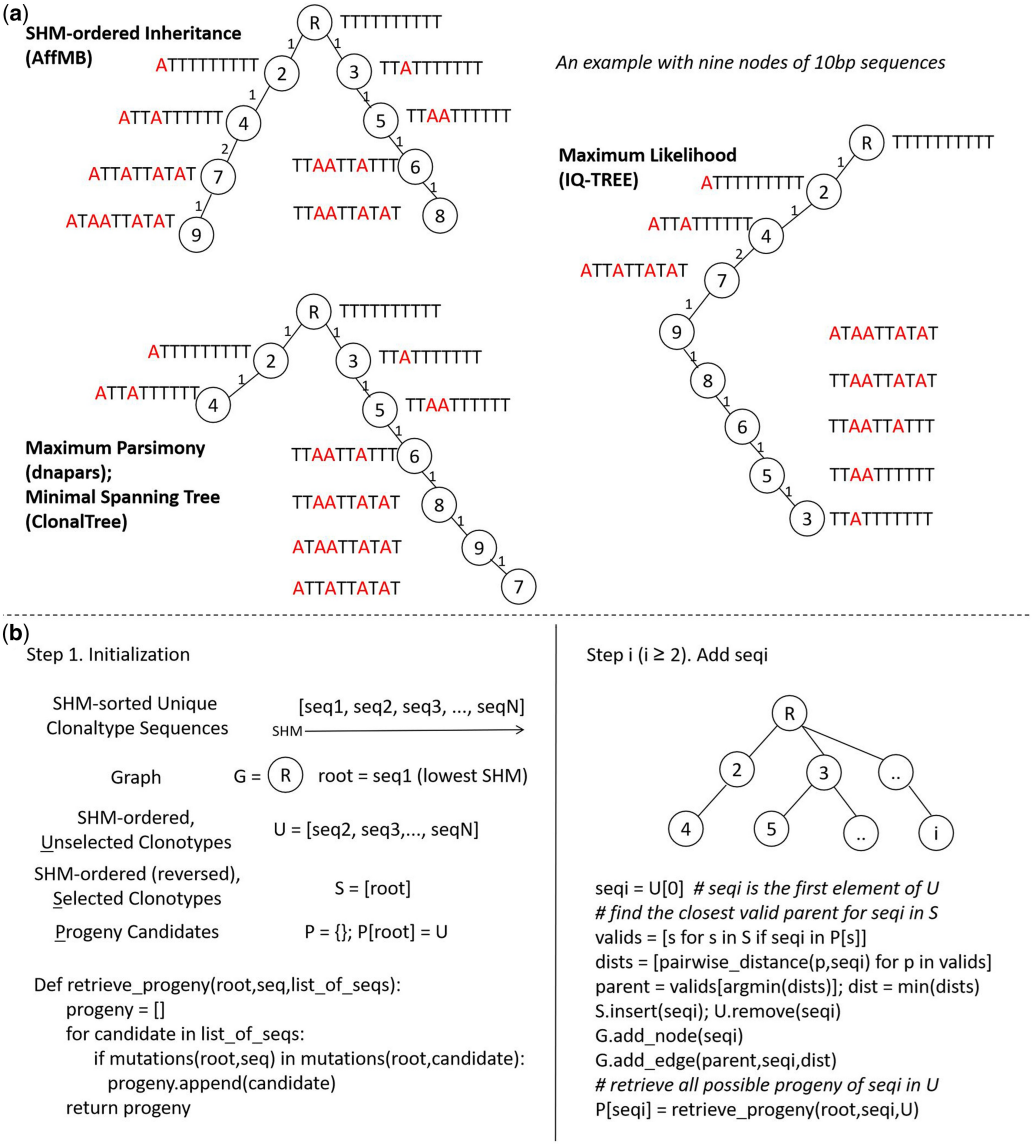
Diagrams illustrating the SHM-ordered inheritance tree algorithm of AffMB. (a) A simple example with nine 10 bp sequences to demonstrate differential behaviour of tree-building algorithms. A tree of each algorithm is generated by running a representative software that implements the algorithm: AffMB for SHM-ordered inheritance tree, dnapars for maximum parsimony tree, ClonalTree for MST, and IQ-tree for maximum likelihood tree. Nodes in all trees are first numbered according to the number of mutated positions (i.e. SHM rate), then sequences with identical SHM rates are further ordered alphabetically. (b) A diagram illustrating the steps to build an SHM-ordered inheritance tree.

In this context, we identified areas where existing tools for Ig/B-cell lineage tree construction could be further improved. First, current algorithms generally fail to guarantee the inheritance of advantageous mutations from parent to child. As illustrated by the example shown in Fig. 1a, one mutation is uninherited (node 9 to node 7) in both the maximum parsimony tree (Felsenstein 2005) and the MST (Abdollahi *et al.* 2023), while multiple mutations are lost (node 9 to 8, 8 to 6, 6 to 5, and 5 to 3) in the maximum likelihood tree (Trifinopoulos *et al.* 2016). Second, existing algorithms ignore the directionality of SHM accumulation. A biologically plausible Ig/B-cell lineage tree should depict key mutation trajectories that progressively increase affinity and divergence (i.e. SHM rate). While positive SHM accumulation from parent to child is expected, the example shown in Fig. 1a demonstrates negative accumulation in the maximum parsimony tree, the MST, and the maximum likelihood tree. Third, most (if not all) Ig/B-cell lineage tree tools (Barak *et al.* 2008, DeWitt *et al.* 2018, Yang *et al.* 2020, Abdollahi *et al.* 2023) require single-lineage input and a known germline sequence. However, BCR sequencing data typically comprises repertoires with thousands of lineages (clonal groups). Adapting these tools for repertoire analysis requires additional scripting for clonal grouping and root inference, creating usability barriers for non-expert users. Although tools such as Dowser (Hoehn *et al.* 2022) can process repertoire input using external packages for clonal grouping, their output trees remain binary phylogenetic trees.

**Figure 2.**
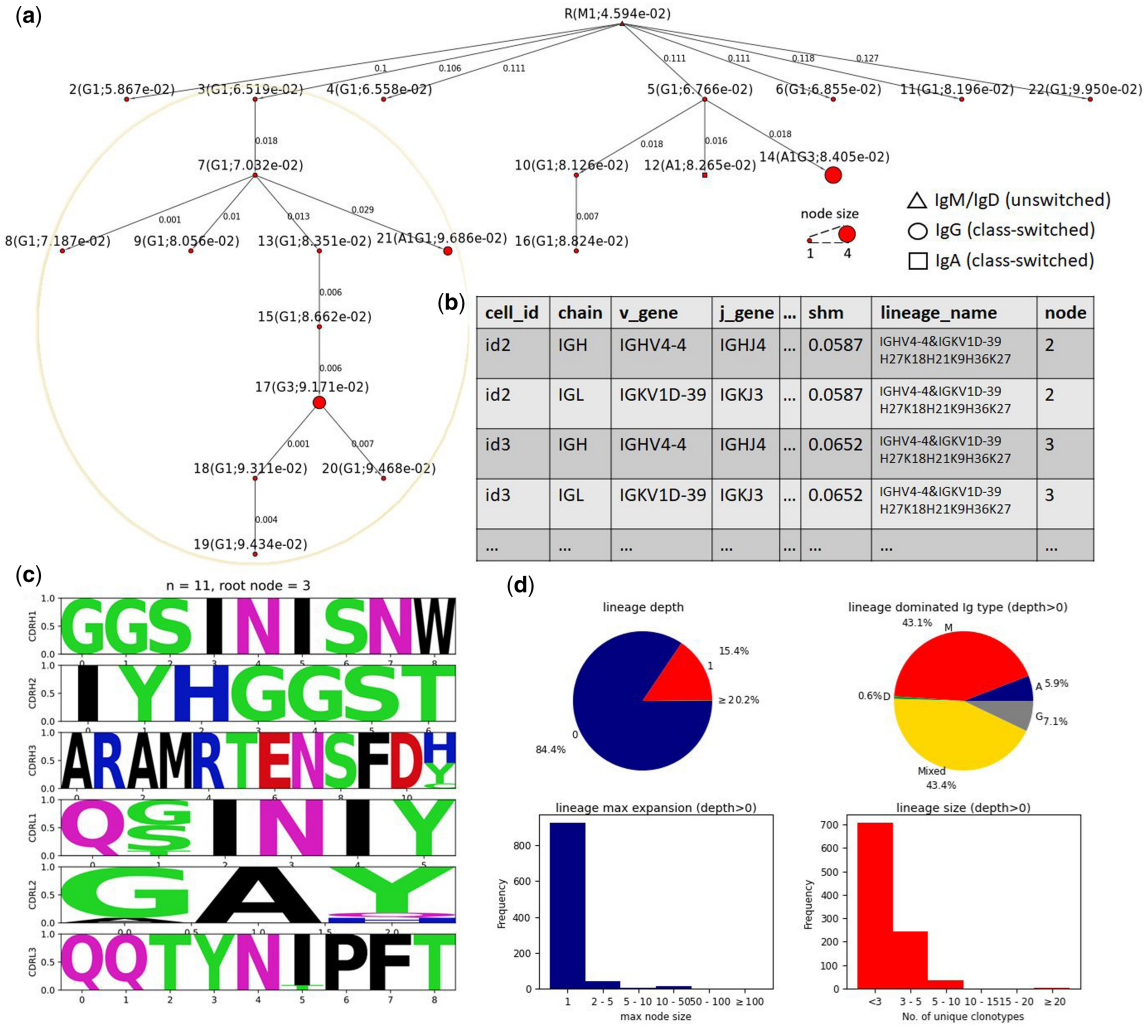
Overview of AffMB output. (a) An example B-cell lineage tree figure generated by AffMB. Each node represents a unique clonotype. Annotations for each node start with node name (e.g. R standing for root node), followed by isotype and node size information (e.g. G2 means the genotype is shared by two IgG B cells; A1G1 means the genotype accounts for one IgA and one IgG cells) and SHM rate (e.g. the SHM rate of the root node R is 4.594×$10^{-2}$). The shape of the nodes is dictated by isotype: triangle for the unswitched isotypes IgM or IgD, circle for IgG, and rectangle for IgA. The size of the node is proportional to node size (i.e. the number of cells sharing the genotype). Edges of the tree are labelled with normalized pairwise edit distance of the nodes, defined as the pairwise Levenshtein distance divided by the length of the parent node sequence. Beige oval highlights a deep branch with closely related nodes, indicating strong signs of affinity maturation. The highlighted branch of interests can be further visualized with logo plots as shown in (d). (b) A schematic representation of the csv table output by AffMB. The table provides cell-to-node mapping to the lineage tree figures. Some columns are omitted due to limited space. (c) An example of logo plots at the amino acid level for a branch of interest highlighted with beige oval in (a). Nucleotide-level logo plots can also be generated as an option. (d) Lineage statistics (see Section 2) summary plots on lineage depth, dominant isotype of lineages, the extent of clonal expansion, and the lineage size. Single-node lineages have zero depth, so depth > 0 means excluding the single-node lineages.

To address the limitations, we developed AffMB (Affinity Maturation of B-cell receptor), a Python package designed for repertoire-level analysis and visualization of affinity maturation through the construction of SHM-ordered, inheritance-based B-cell lineage trees (Fig. 2). AffMB supports full-length single-cell and bulk BCR repertoire data, offering flexible automation for generating, filtering, and visualizing lineage trees of interest. We validated and benchmarked AffMB’s tree-building algorithm using real-world and simulated datasets. Furthermore, we demonstrated AffMB’s utility in evaluating individual immunization responses and explored its potential for identifying high-affinity antibody candidates from single-cell BCR sequencing (scBCR-seq) data (Brewer *et al.* 2022).

## 2 Materials and methods

AffMB is designed for BCR repertoire analysis. A BCR repertoire comprises multiple lineages (clonal groups), where each lineage represents a cluster of closely related BCRs that are expected to share a common ancestor. For an input repertoire, AffMB first identifies lineages by grouping their V gene usage and lengths of all CDRs. For each lineage, AffMB then reconstructs an SHM-ordered inheritance tree to infer Ig gene evolution trajectories that progressively increase both affinity and SHM rate. Additionally, AffMB generates comprehensive lineage statistics, including lineage depth, size, dominant isotypes, and clonal expansion levels.

The following subsections detail each step and the tree-building algorithm implemented in AffMB.

### 2.1 Input formats

AffMB accepts two types of input: a raw contig file in FASTA format or a processed AIRR rearrangement file in TSV format. The AIRR rearrangement TSV file must at least contain the following necessary fields: sequence_id, sequence, productive, locus, v_call, j_call, sequence_alignment, germline_alignment, fwr1, fwr1_aa, cdr1, cdr1_aa, fwr2, fwr2_aa, cdr2, cdr2_aa, fwr3, fwr3_aa, cdr3, cdr3_aa, fwr4, and fwr4_aa. For a raw input in FASTA format, AffMB offers an API function that calls an external V(D)J annotation tool IgBLAST (Ye *et al.* 2013), to generate the required AIRR rearrangement file.

### 2.2 Clonal grouping to form lineages

The first step of AffMB is to group the cell/sequence in the input BCR repertoire into lineages. For a single-cell input, cells sharing identical V gene usage plus identical CDR1, CDR2, and CDR3 lengths in both heavy and light chains are grouped together as a lineage. The same rule applies to bulk input, except only considering the heavy chain. While most CDR mutations are substitutions, insertions or deletions (indels) occur at much lower frequencies. Under these stringent criteria, sequences with CDR indels will form separate lineages due to differing CDR lengths. Alternatively, users may opt for looser criteria, grouping sequences with matching V genes and ≤3 total CDR length differences, resulting in fewer but larger lineages. However, caution is warranted when interpreting multiple indels within a lineage, as some may reflect distinct origins rather than true indels, given the expected low indel rate.

### 2.3 Clonotype and node definition

A full-length BCR sequence encompasses the variable (V) region of a BCR chain, comprising seven distinct regions: three complementarity-determining regions (CDRs 1–3) interspersed with four framework regions (FWRs 1–4). The CDRs constitute the critical antigen-binding domains of an antibody. In AffMB’s lineage trees, each ‘node’ represents a unique ‘clonotype’**—**defined as either: (i) a unique V-region nucleotide sequence corresponding to a population of identical cells (Sofou *et al.* 2023), or (ii) alternatively, the concatenated nucleotide sequence of all CDRs (hereafter denoted as full CDR). The former provides complete sequence-level resolution required for antibody reconstruction. At the same time, the latter focuses specifically on CDR-level selection patterns, highlighting evolutionary changes in the most functionally significant regions, and increasing the speed. We recommend using both to obtain complementary insights into affinity maturation dynamics.

### 2.4 Calculation of SHM rate

AffMB calculates SHM rates separately for heavy (hSHM) and light (lSHM) chains by determining the mutation fraction between each V-region nucleotide sequence and its inferred germline counterpart. For single-cell data, users may select from multiple SHM rate calculation methods: hSHM alone, the maximum of hSHM and lSHM, or their mean (default option). When clonotypes are defined by full CDR (rather than complete V-region), a single clonotype may encompass multiple V-region nucleotide sequences with varying SHM rates. In such cases, the node’s SHM rate represents the mean value across all cells/sequences sharing that clonotype.

### 2.5 SHM-ordered inheritance tree

Figure 1a compares AffMB’s SHM-ordered inheritance tree with conventional tree algorithms, including maximum parsimony [represented by dnapars (Felsenstein 2005)], maximum likelihood [represented by IQ-TREE (Trifinopoulos *et al.* 2016)], and minimal spanning tree [represented by ClonalTree (Abdollahi *et al.* 2023)]. The SHM-ordered tree exhibits two defining features: (i) root-to-tip SHM rate ordering, and (ii) complete inheritance of parental forward mutations by child nodes. These properties model affinity maturation dynamics, where B cells progressively accumulate beneficial mutations through SHM while increasing their divergence (reflected by rising SHM rates).

Unlike the conventional sense of evolution, where environmental changes over extended timescales may permit backward evolution (i.e. reversed mutations), affinity maturation occurs rapidly under constant antigenic selection. With selective pressure intensifying through successive generations due to clonal competition, reversion mutations are not typically expected to occur. Given the stable mutation rate of 1–2 mutations per generation (Teng and Papavasiliou 2007), neutral mutations are evolutionarily costly as they represent missed opportunities for affinity enhancement. Consequently, only forward mutations that improve antigen binding are expected to persist in the population. Crucially, since sequenced BCRs represent selection survivors, the SHM-ordered tree reconstructs the key mutational trajectories leading to high-affinity antibody production.

Figure 1b illustrates the steps of AffMB’s SHM-ordered inheritance tree algorithm for constructing a B-cell lineage tree. As described above, each node represents a unique clonotype within a lineage. The algorithm begins by sorting all unique clonotype sequences in the lineage by their SHM rates. The clonotype with the lowest SHM rate (least divergent) is selected as the root node. When multiple candidates share the lowest SHM rate, priority is given to clonotypes with unswitched isotypes (IGHM/IGHD); if ties remain, a dummy root node is created by introducing ‘D’ bases at ambiguous positions (e.g. combining ‘AGC’ and ‘CGC’ yields ‘DGC’). The algorithm initializes a directed graph G with this root node, along with: (i) a list S containing selected clonotypes, (ii) a list U of SHM-sorted unselected clonotypes, and (iii) a dictionary P recording clonotype-progeny relationships. Possible progeny are identified when clonotypes in U share all CDR mutations with a parent in S, with inheritance validity determined solely by CDR mutations (regardless of clonotype definition) due to the selection priority of CDRs. Initially, all elements in U are potential progeny of the root. The algorithm then iteratively: (i) selects the first clonotype in U, (ii) connects it to its closest valid parent (smallest edit distance) in S, (iii) transfers it from U to S (maintaining reverse SHM order in S), and (iv) updates P with its potential progeny, until U is exhausted (Fig. 1b).

AffMB constructs an SHM-ordered inheritance tree for each lineage (Fig. 2a), where each node represents a unique clonotype. The ‘node size’ corresponds to clonotype abundance (cell/sequence count). Directed edges connect parent–child nodes, with ‘edge weights’ showing normalized pairwise edit distance, defined as the pairwise Levenshtein distance divided by the length of the parent node sequence. Lineages containing only one unique clonotype (single-node lineages with zero depth) may represent either naive cells or distant memory cells unlikely to participate in recent/ongoing affinity maturation, as their ancestral/descendant relationships remain unobserved in sequencing data. These single-node lineages are consequently excluded from downstream analysis.

### 2.6 Lineage statistics

AffMB generates comprehensive statistical summaries for each input repertoire, including: (i) lineage depth, defined as the edge count along the longest path in the lineage tree; (ii) dominant isotype classification, where lineages with >50% nodes sharing an isotype are considered isotype-dominated, otherwise classified as isotype-mixed; (iii) clonal expansion level, measured by the maximum node size within the lineage; and (iv) lineage size, representing the number of nodes (i.e. number of unique clonotypes).

### 2.7 Visualization of a branch of interests

AffMB automatically identifies deep (default depth ≥2) and large (default size ≥5) branches of closely related nodes in each lineage tree. As previously defined, edge weights represent normalized pairwise edit distances between nodes. Users may adjust the edit distance threshold (default ≤0.1) to control branch sizes by modifying the criteria for node relatedness. For qualifying branches, AffMB generates both amino acid and nucleotide logo plots using Logomaker (Tareen and Kinney 2020) to visualize mutation patterns.

### 2.8 Simulation of lineage trees and benchmarking

To benchmark our SHM-ordered inheritance tree algorithm, we developed a simulation subprogram that generates synthetic lineage trees. The simulation begins with a randomly generated germline sequence of length *L* as the root. Child nodes are then recursively generated under the following constraints: (i) the number of children and mutations per node follow Poisson distributions with means $\lambda_{c}$ and $\lambda_{m}$, respectively; (ii) 10% of positions are randomly designated as mutation hotspots with 3× higher mutation probability; (iii) all parental mutations are inherited (no reversed mutations allowed); and (iv) tree growth terminates when either the total node count exceeds *N* or 95% of positions have mutated. We tested parameter combinations covering biologically relevant ranges: L∈{100,200} approximating single/paired-chain full CDR lengths, N∈{100,200} enabling large, complex trees, $\lambda_{m}\in \{1,2\}$ matching observed 1–2 mutations/generation (Teng and Papavasiliou 2007), and $\lambda_{c}\in \{2,3,4\}$ corresponding to an average of three cell cycles per germinal centre B-cell in the dark zone (Young and Brink 2021). This 2 × 2 × 2 × 3 parameter space was repeated 50 times per combination, yielding 1200 trees for comprehensive benchmarking.

We evaluated tree reconstruction performance against two state-of-the-art B-cell/Ig lineage tree methods: ClonalTree (Abdollahi *et al.* 2023) and GLaMST (Yang *et al.* 2020), both run with default parameters. Graph edit distances between simulated and reconstructed trees were computed using GMatch4py: https://github.com/jacquesfize/GMatch4py. GLaMST creates additional inferred intermediate nodes. To ensure fair comparisons, we removed all inferred nodes and reconnected the sampled nodes accordingly to avoid a large graph edit distance caused by these extra nodes. All benchmarks were executed on a notebook PC (13th Gen Intel i5-1340P, 1.90 GHz; 16GB RAM, 3200 MHz) under single-threaded conditions.

## 3 Results

Figure 2 summarizes AffMB’s output for each input BCR repertoire. The package generates multiple B-cell lineage trees representing potential affinity maturation processes, where nodes (unique clonotypes) are connected by edges weighted with normalized pairwise edit distances and annotated with clonotype abundance, isotype, and SHM rate (Fig. 2a). These trees are constructed using the SHM-ordered inheritance tree algorithm (see Section 2). AffMB outputs a folder which includes: (i) individual lineage tree figures, (ii) a CSV table mapping node positions to input file entries (Fig. 2b), (iii) logo plots at amino acid/nucleotide level highlighting potential deeply evolved branches (Fig. 2c) and (iv) a lineage statistic summary figure for the input repertoire (Fig. 2d). Optionally, users can set a tree depth threshold (default = 2) to focus on lineages that demonstrate patterns of affinity maturation. The logo plots are options provided to users to automatically identify antibody candidates, which is expected in deep and expanded branches of closely related nodes (e.g. the 11-node cluster highlighted in Fig. 2a).

We validated the methodology of AffMB on several aspects. We confirmed that the SHM-ordered inheritance trees implemented in AffMB achieve complete 100% SHM ordering, and that SHM is not necessarily ordered in baseline MST trees, with 83.5%–100% SHM ordering on a BNT162b2 vaccination scBCR-seq dataset (*n* = 42) (Brewer *et al.* 2022) (Fig. 1 and Supplementary Methods, available as supplementary data at *Bioinformatics* online). Then we evaluated tree reconstruction performance using 1200 simulated trees, comparing AffMB against two state-of-the-art approaches: ClonalTree (Abdollahi *et al.* 2023) and GLaMST (Yang *et al.* 2020). AffMB demonstrated superior speed and, in most parameter configurations, achieved the lowest average graph edit distance (Table 1). Finally, analysis of the BNT162b2 dataset (*n* = 42) using AffMB demonstrated tight clonal relationships, with a mean edge edit distance of 0.07 and 99% of edges <0.17 at the sequence level (Fig. 2, available as supplementary data at *Bioinformatics* online), supporting the validity of AffMB’s clonal grouping.

**Table 1.** Simulation results for various parameters.^a^

Parameters					Graph edit distance			Speed (s)		
SeqL	N_nodes	λ c	λ m	N_trees	AffMB	ClonalTree	GLaMST	AffMB	ClonalTree	GLaMST
100	100	2	1, 2	100	2.50	3.08	5.76	0.11	0.61	148.25
100	100	3	1, 2	100	2.42	2.50	4.99	0.09	0.63	44.66
100	100	4	1, 2	100	2.68	2.76	5.83	0.08	0.64	21.45
100	200	2	1, 2	100	30.52	39.57	58.51	0.51	7.90	3131.38
100	200	3	1, 2	100	18.54	24.17	37.96	0.47	7.98	1221.20
100	200	4	1, 2	100	12.90	15.64	25.94	0.41	8.06	565.40
200	100	2	1, 2	100	0.68	0.66	1.30	0.13	0.65	49.96
200	100	3	1, 2	100	0.78	0.80	1.76	0.11	0.65	23.70
200	100	4	1, 2	100	1.38	1.36	3.04	0.09	0.66	19.14
200	200	2	1, 2	100	2.71	3.00	5.33	0.70	8.04	2576.52
200	200	3	1, 2	100	2.06	2.08	4.20	0.58	8.14	547.77
200	200	4	1, 2	100	2.66	2.64	5.94	0.49	8.17	200.44

a Best performances (smallest average graph edit distance) are underlined. All benchmarks were executed on a notebook PC (13th Gen Intel i5-1340P, 1.90 GHz; 16GB RAM, 3200 MHz) under single-threaded conditions.

Next, we analysed the BNT162b2 vaccination dataset (Brewer *et al.* 2022) of scBCR-seq repertoire samples (*n* = 42) using AffMB. The dataset contains three positive control samples, three negative control samples, and 36 samples from nine healthy individuals (P1–P9) who received the BNT162b2 vaccine and sampled at multiple time points: pre-vaccination (Day 0), 7–9 days post-first dose (Day 7), 21–23 days post-first dose (Day 21; second dose administration), and 28 days post-first dose (Day 28). The positive control samples consisted of FACS-sorted S-tetramer double-positive cells, while the negative control samples contained S-tetramer double-negative cells. The following subsections detail our analytical findings.

### 3.1 Large, deep, and expanded lineages found in positive but not in negative controls

**Figure 3.**
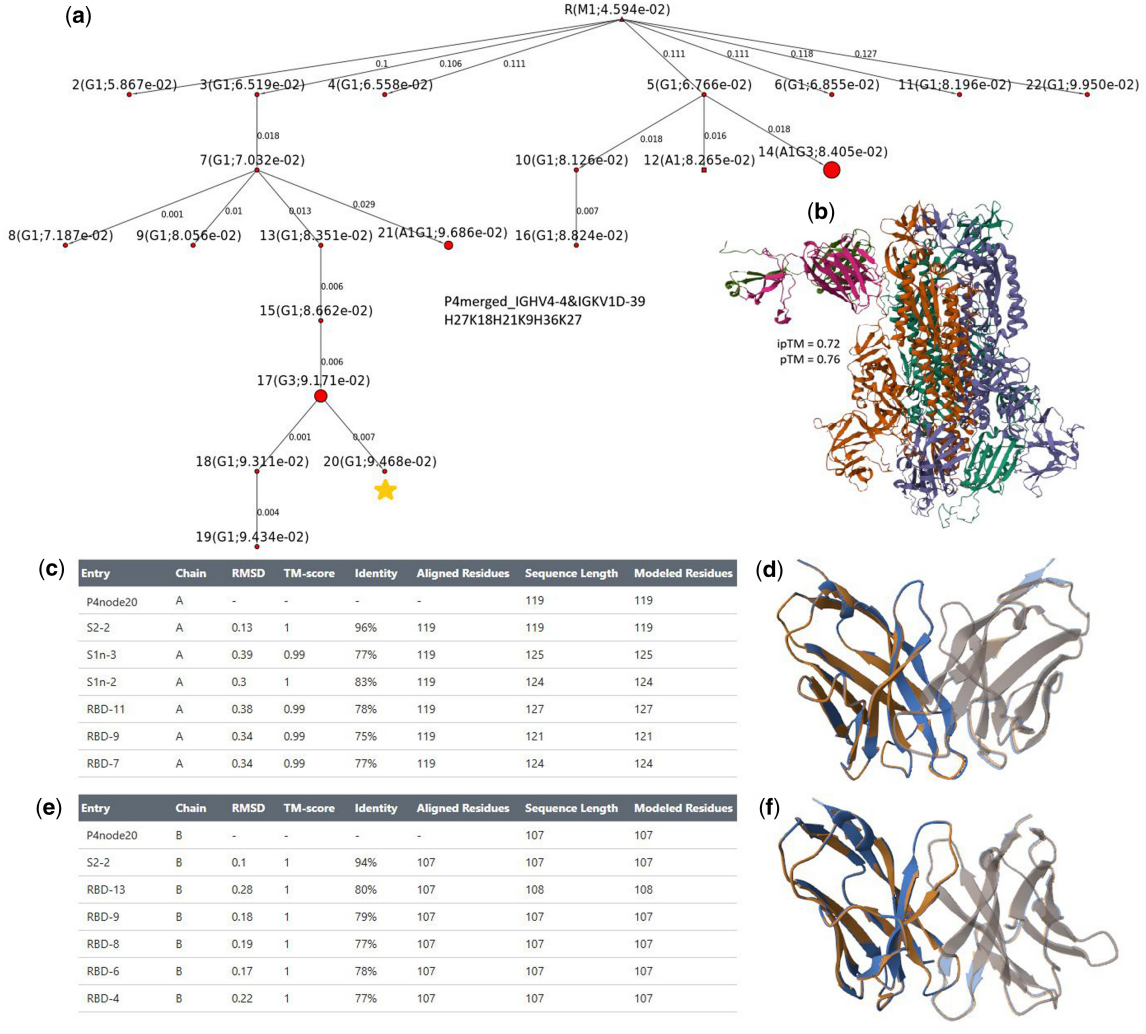
An example of an identified candidate with high structural and sequence similarity to an experimentally validated high-affinity antibody. Lineage tree is named by sample name followed by VH gene and VL gene name, and finally the length of each CDR with the order: CDRH1, CDRL1, CDRH2, CDRL2, CDRH3, CDRL3. (a) The large, deep, and expanded lineage tree from P4, where the candidate (node 20, highlighted with a star) was selected. (b) AlphaFold3 prediction of the interaction between the candidate and the SARS-CoV-2 Spike protein trimer, with predicted binding site at the S2 subunit. (c) Pairwise structural alignment results of the candidate and the top matches on heavy chain (chain A). (d) Visualization of the alignment between the candidate and the S2-2 antibody (top match) on the heavy chain. (e) Pairwise structural alignment results of the candidate and the top matches on light chain (chain B). (f) Visualization of the alignment between the candidate and the S2-2 antibody (top match) on the light chain.

We observed distinct lineage tree characteristics in positive versus negative control samples. Negative control B cells exhibited minimal affinity maturation patterns, with almost no deep trees (tree depth ≥2) detected across all negative control samples (Fig. 3, available as supplementary data at *Bioinformatics* online, bottom left grey tree). Conversely, positive controls consistently displayed robust affinity maturation patterns, characterized by large, deep, and clonally expanded lineages in every positive control sample (Fig. 3, available as supplementary data at *Bioinformatics* online, red trees).

### 3.2 Lineage statistics reflect immune status

We further analysed lineage statistics across samples and individuals (Fig. 4, available as supplementary data at *Bioinformatics* online). Given the limited B-cell capture per time-point, individual time-point samples reflect real-time status but may miss continuous response dynamics. Pre-vaccination (Day 0) revealed heterogeneous baseline states: individuals P1, P3, and P4 showed resting states with minimal affinity maturation patterns, while P2 and P5–P9 exhibited active states with deep lineages (depth≥2) already, suggesting recent immune stimulation. In initially resting individuals (P1, P3, P4), response intensity peaked sharply after the first dose (Day 0–Day 7), whereas initially active individuals showed variable patterns.

To reconstruct comprehensive affinity maturation trajectories throughout the whole sampling period, we merged all time points per individual. After merging the samples, deep lineages are observed in all individuals, despite P1 and P3 maintained significantly fewer deep lineages. Notably, P1 later contracted COVID-19 post-vaccination (Brewer *et al.* 2022), suggesting a potential link between lineage statistics and clinical outcomes.

### 3.3 Identifying candidates for high-affinity antibodies

Having observed the distinctive characteristics in positive versus negative controls, we systematically identified large (number of nodes ≥8), deep (tree depth ≥2), and clonally expanded (max node size ≥2) lineages in the merged samples as potential sources of neutralizing antibody candidates, focusing on nodes with the highest SHM rates within the deepest branches. Several candidate sequences exhibited high structural and/or sequence similarity to experimentally validated high-affinity S2^+^ and RBD^+^ antibodies (see Supplementary Methods, available as supplementary data at *Bioinformatics* online). For instance, a lineage (IGHV4-4/IGKV1D-39) from the individual P4 displayed substantial size (22 nodes), depth (tree depth = 7), and moderate clonal expansion (Fig. 3a). The selected candidate, node 20 (highlighted with a star), was predicted to bind the S2 subunit by AlphaFold3 (Fig. 3b) (Abramson *et al.* 2024). Structural alignment (Bittrich *et al.* 2024) confirmed close similarity to known S2-targeting antibody S2-2 (Fig. 3c–f and Table 1, available as supplementary data at *Bioinformatics* online). Similarly, a lineage (IGHV4-39/IGKV3-15) from P8 showed robust affinity maturation patterns (8 nodes, tree depth = 3, large expansion; Fig. 5a, available as supplementary data at *Bioinformatics* online), where candidate node 8 was predicted RBD-binding by AlphaFold3 (Fig. 5b, available as supplementary data at *Bioinformatics* online) and demonstrated structural homology to RBD-targeting antibody RBD-11 by structural alignment (Fig. 5c–f and Table 2, available as supplementary data at *Bioinformatics* online). These findings validate our approach for identifying antibody candidates through comprehensive lineage analysis.

## 4 Discussion

We present AffMB, a comprehensive toolkit for analysing affinity maturation through constructions of SHM-ordered, inheritance-based B-cell lineage trees. The SHM-ordered inheritance tree demonstrates biological validity by enforcing parent-to-child inheritance patterns with positive SHM accumulation. Our application analysis revealed that large, deep, and clonal expanded lineages identified by AffMB frequently contain nodes with high structural similarity to known high-affinity antibodies, establishing the method’s potential for discovering clinically relevant antibody candidates.

AffMB models affinity maturation as a biological optimization process, where B cells evolve from minimally mutated, low-affinity states to highly divergent, high-affinity antibody producers. Under SHM and strong selective pressure, only B cells inheriting and acquiring favourable mutations gain survival advantages. This evolutionary constraint ensures that surviving (observed) B cells predominantly exhibit inherited beneficial mutations, resulting in progressive divergence accumulation. By introducing the SHM rate as a key feature, AffMB uses SHM ranking as a proxy for affinity ranking, leveraging the established biological correlation between SHM and affinity. Under these biological constraints, the SHM-ordered inheritance tree algorithm aims to reconstruct the most plausible affinity optimization trajectories from root to tips, where increasing SHM rates reflect the natural selection-driven improvement of antibody affinity.

We demonstrated AffMB’s capability to track affinity maturation in individual samples and identify high-affinity antibody candidates. By monitoring affinity maturation dynamics, we can infer the strength of an individual’s immune response to antigens or vaccines, thereby evaluating therapeutic efficacy. Direct discovery of antibody candidates from human sequencing data offers potential advantages over current protein engineering approaches, such as directed evolution, which artificially mimics affinity maturation through iterative mutagenesis and selection cycles. While rational design strategies incorporating prior knowledge (e.g. site-directed mutagenesis and computational modelling) can help reduce library sizes for screening (Kim *et al.* 2023), these methods remain expensive, time-consuming, and labour-intensive. Additionally, since these processes occur outside the human system, they face substantial challenges in antibody humanization to reduce immunogenicity while preserving affinity. In contrast, direct extraction of human antibody candidates from natural BCR repertoire sequences inherently reduces immunogenicity concerns and provides a rich library of therapeutically relevant antibody prototypes, leveraging the body’s own optimized selection process.

## Supplementary Material

btaf346_Supplementary_Data

## Data Availability

All the data used within this study can be retrieved from public databases. The source code of this study is available at Zenodo: https://doi.org/10.5281/zenodo.15463545. AffMB is an open-source Python package with source code freely available at https://github.com/deepomicslab/AffMB.

## References

[btaf346-B1] Abdollahi N , Jeusset L, de Septenville A et al Reconstructing B cell lineage trees with minimum spanning tree and genotype abundances. BMC Bioinformatics 2023;24:70–19. 36849917 10.1186/s12859-022-05112-z PMC9972711

[btaf346-B2] Abramson J , Adler J, Dunger J et al Accurate structure prediction of biomolecular interactions with AlphaFold 3. Nature 2024;630:493–500. 38718835 10.1038/s41586-024-07487-w PMC11168924

[btaf346-B3] Barak M , Zuckerman NS, Edelman H et al IgTree©: creating immunoglobulin variable region gene lineage trees. J Immunol Methods 2008;338:67–74. 18706908 10.1016/j.jim.2008.06.006

[btaf346-B4] Bittrich S , Segura J, Duarte JM et al RCSB protein data bank: exploring protein 3D similarities via comprehensive structural alignments. Bioinformatics 2024;40:3–6. 10.1093/bioinformatics/btae370 PMC11212067 38870521

[btaf346-B5] Brewer RC , Ramadoss NS, Lahey LJ et al BNT162b2 vaccine induces divergent B cell responses to SARS-CoV-2 S1 and S2. Nat Immunol 2022;23:33–9. 34848871 10.1038/s41590-021-01088-9 PMC8776031

[btaf346-B6] DeWitt WS , Mesin L, Victora GD et al Using genotype abundance to improve phylogenetic inference. Mol Biol Evol 2018;35:1253–65. 29474671 10.1093/molbev/msy020 PMC5913685

[btaf346-B7] Felsenstein J. 2005. PHYLIP (phylogeny inference package). Version 3.6. Produced and distributed by author. https://evolution.genetics.washington.edu/phylip.html (9 June 2025, date last accessed).

[btaf346-B8] Hoehn K , Kleinstein S. B cell phylogenetics in the single cell era. Trends Immunol 2024;45:62–74. 38151443 10.1016/j.it.2023.11.004 PMC10872299

[btaf346-B9] Hoehn K , Pybus O, Kleinstein S. Phylogenetic analysis of migration, differentiation, and class switching in B cells. PLoS Comput Biol 2022;18:e1009885. 35468128 10.1371/journal.pcbi.1009885 PMC9037912

[btaf346-B10] Kim J , McFee M, Fang Q et al Computational and artificial intelligence-based methods for antibody development. Trends Pharmacol Sci 2023;44:175–89. 36669976 10.1016/j.tips.2022.12.005

[btaf346-B11] Sofou E , Vlachonikola E, Zaragoza-Infante L et al Clonotype definitions for immunogenetic studies: proposals from the EuroClonality NGS Working Group. Leukemia 2023;37:1750–2. 37391484 10.1038/s41375-023-01952-7 PMC10400411

[btaf346-B12] Tareen A , Kinney JB. Logomaker: beautiful sequence logos in Python. Bioinformatics 2020;36:2272–4. 31821414 10.1093/bioinformatics/btz921 PMC7141850

[btaf346-B13] Teng G , Papavasiliou N. Immunoglobulin somatic hypermutation. Annu Rev Genet 2007;41:107–20. (), 17576170 10.1146/annurev.genet.41.110306.130340

[btaf346-B14] Trifinopoulos J , Nguyen L-T, von Haeseler A et al W-IQ-TREE: a fast online phylogenetic tool for maximum likelihood analysis. Nucleic Acids Res 2016;44:W232–35. 27084950 10.1093/nar/gkw256 PMC4987875

[btaf346-B15] Yang X , Tipton CM, Woodruff MC et al GLaMST: grow lineages along minimum spanning tree for B cell receptor sequencing data. BMC Genomics 2020;21:583–11. 32900378 10.1186/s12864-020-06936-w PMC7488003

[btaf346-B16] Ye J , Ma N, Madden TL et al IgBLAST: an immunoglobulin variable domain sequence analysis tool. Nucleic Acids Res 2013;41:W34–40. 23671333 10.1093/nar/gkt382 PMC3692102

[btaf346-B17] Young C , Brink R. The unique biology of germinal center B cells. Immunity 2021;54:1652–64. 34380063 10.1016/j.immuni.2021.07.015

