# Supplementary material for: AffMB: affinity maturation analysis with SHM-guided B-cell lineage trees
Source: Bioinformatics. 2025 Jul 17;41(7):btaf346. doi: 10.1093/bioinformatics/btaf346 (PMC12282942; doi:10.1093/bioinformatics/btaf346)
Supplement: btaf346_Supplementary_Data [file btaf346_supplementary_data.pdf]

Supplementary Methods

Validation of SHM ordering

To validate that AffMB's SHM-ordering inheritance tree achieves better SHM ordering than baseline, we calculated proportion of edges that lead to increase in SHM. For each edge, we calculate the difference of SHM rates between the parent and the child node connected by the edge. If the difference is non-negative, the edge is an SHM-ascending edge; otherwise if the difference is negative, the edge is an SHM-descending edge (Supplementary Figure 1). The SHM ordering consistency of a sample is defined as the number of SHM-ascending edges devided by total number of edges in all trees. For baseline, we implemented Prim's MST which went through the same process of root selection (root as the starting vertex) and clonotype-sorting by SHM rates in the unselected clonotypes as in the SHM-ordering inheritance tree. The SHM ordering consistency of SHM-ordering inheritance trees were compared with the MSTs in the 42 single-cell samples used in this study (Supplementary Figure 1). SHM-ordering inheritance tree achieves 100% SHM ordering consistency regardless of clonotype definition. MST showed 83.5% - 100% SHM ordering consistency depending on samples and clonotype definition.

Protein structural alignment and S-binding prediction of candidate antibody sequences

The BNT162b2 study[1] performed experimental validation on spike protein reactivity of 50 selected candidate antibodies. We retrieved the paired V_H and V_L sequences of the 50 expressed monoclonal antibodies with reactivity to each subunit of the spike protein assessed by ELISA. Within these antibodies, we selected those with AUC>3 as positive binders, resulting in a total of 24 S^+ antibodies (Supplementary Table 1).

We used Igfold[2] to generate the predicted structures of the antibodies and candidates (.pdb format). The generated protein structures were aligned using the online pairwise 3D structure alignment tool[3] by RCSB Protein Data Bank (PDB). Alignment results and scores of the two example candidates against the 24 S^+ antibodies are detailed in Supplementary Table 1, with the top match antibody highlighted in red. Since both chains must be considered, the top match is not necessarily the closest match in a single chain but must be the closest overall, at least with RMSD < 0.5 in both chains.

We obtained the amino acid sequence of the SARS-CoV2 spike protein[4] (open conformation) using the PDB ID 7zh5. For each candidate, the heavy chain sequence consisted of the V_H concatenated by the C_H sequence, the light chain sequence consisted of the V_L concatenated by the C_L sequence. If the candidate has an unswitched isotype (IgM or IgD), we used the IGHG1 sequence as the C_H sequence. The heavy chain sequence, light chain sequence and the spike protein sequence (with parameter set to 3-copies because the protein is usually a trimer) were input to AlphaFold3 server[5] as separate entries. The predicted results were downloaded from the server and visualized using the online Mol* viewer[6].

References

1. Brewer, R. C., Ramadoss, N. S., Lahey, L. J., Jahanbani, S., Robinson, W. H., & Lanz, T. V. (2022). BNT162b2 vaccine induces divergent B cell responses to SARS-CoV-2 S1 and S2. Nature immunology, 23(1), 33–39.
2. Ruffolo, J. A., Chu, L. S., Mahajan, S. P., & Gray, J. J. (2023). Fast, accurate antibody structure prediction from deep learning on massive set of natural antibodies. Nature communications, 14(1), 2389.
3. Bittrich, S., Segura, J., Duarte, J. M., Burley, S. K., & Rose, Y. (2024). RCSB protein Data Bank: exploring protein 3D similarities via comprehensive structural alignments. Bioinformatics (Oxford, England), 40(6), btae370.
4. Toelzer, C., Gupta, K., Yadav, S. K. N., Hodgson, L., Williamson, M. K., Buzas, D., Borucu, U., Powers, K., Stenner, R., Vasileiou, K., Garzoni, F., Fitzgerald, D., Payré, C., Gautam, G., Lambeau, G., Davidson, A. D., Verkade, P., Frank, M., Berger, I., & Schaffitzel, C. (2022). The free fatty acid-binding pocket is a conserved hallmark in pathogenic β-coronavirus spike proteins from SARS-CoV to Omicron. Science advances, 8(47), eadc9179.
5. Abramson, J., Adler, J., Dunger, J., Evans, R., Green, T., Pritzel, A., Ronneberger, O., Willmore, L., Ballard, A. J., Bambrick, J., Bodenstein, S. W., Evans, D. A., Hung, C. C., O'Neill, M., Reiman, D., Tunyasuvunakool, K., Wu, Z., Žemgulytė, A., Arvaniti, E., Beattie, C., … Jumper, J. M. (2024). Accurate structure prediction of biomolecular interactions with AlphaFold 3. Nature, 630(8016), 493–500.
6. Sehnal, D., Bittrich, S., Deshpande, M., Svobodová, R., Berka, K., Bazgier, V., Velankar, S., Burley, S. K., Koča, J., & Rose, A. S. (2021). Mol* Viewer: modern web app for 3D visualization and analysis of large biomolecular structures. Nucleic acids research, 49(W1), W431–W437.

Supplementary Figures

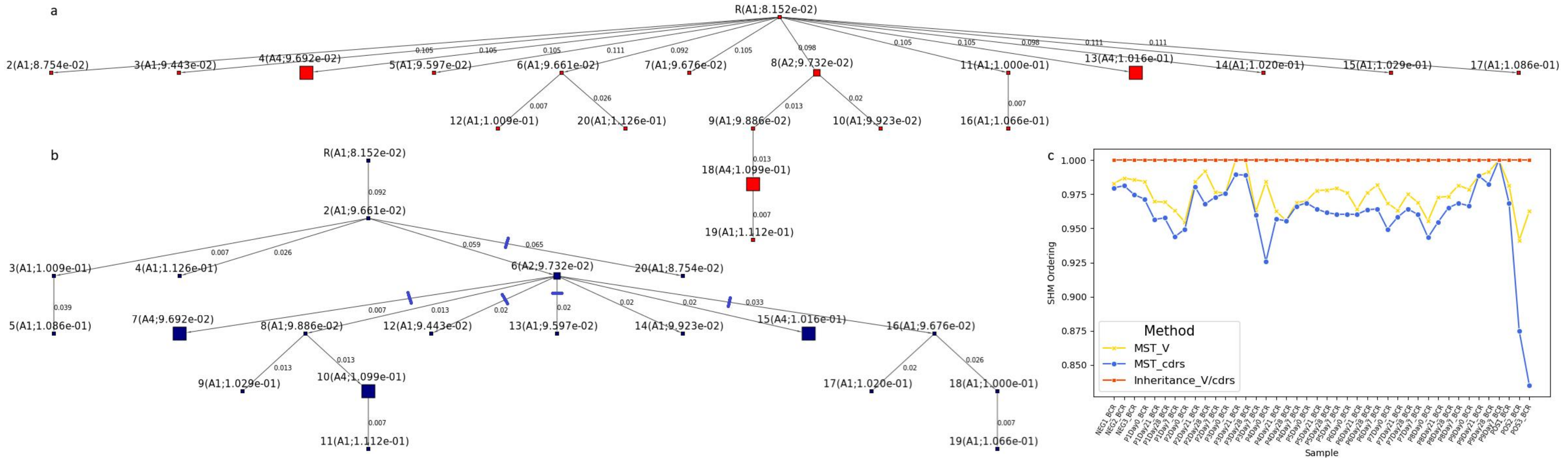

Supplementary Figure 1. Validation of SHM ordering. **a)** An example SHM-ordered inheritance tree from a positive control sample POS1_BCR. Each node represents a unique clonotype of full CDR (i.e., combination of all CDR regions). Annotations for each node starts with node name (e.g., 'R' standing for root node), followed by isotype and node size information and SHM rate. For example, the root node R accounts for one IgA B-cell (A1), with an SHM rate $8.152{\times}10^{-2}$. **b)** A Prim's minimal spanning tree constructed using the BCR sequences in (a). SHM-descending edges are highlighted with hand-written slashes crossing the edge. **c)** A comparison of SHM ordering (see Supplementary Methods) on SHM-ordered inheritance tree (red line) versus Prim's MST (yellow and blue line for sequence and CDR level, respectively) on all samples of the BNT162b2 study[1].

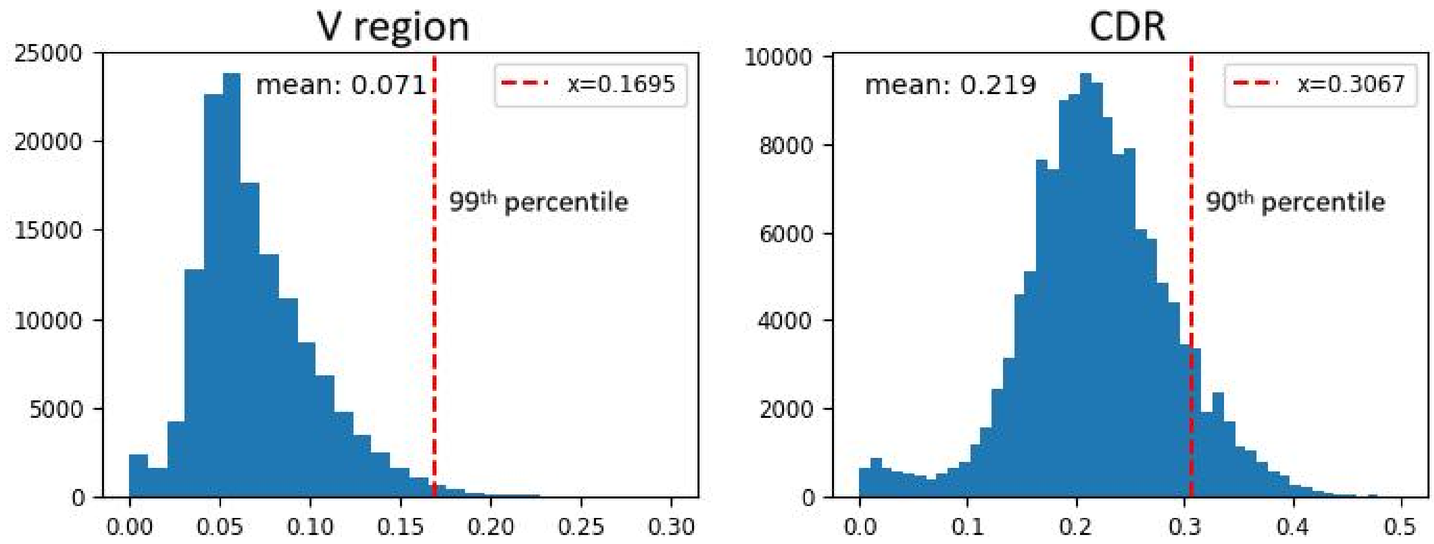

Supplementary Figure 2. Left: histogram showing distribution of edge edit distance at sequence level for the single-cell vaccination data (n=42) from the BNT162b2 study[1]. Red dashed line shows the 99th percentile at 0.1695. Right: histogram showing distribution of edge edit distance at CDR level, 88.5% of edges have an edit distance ≤ 0.3. Red dashed line shows the 90th percentile at 0.3067.

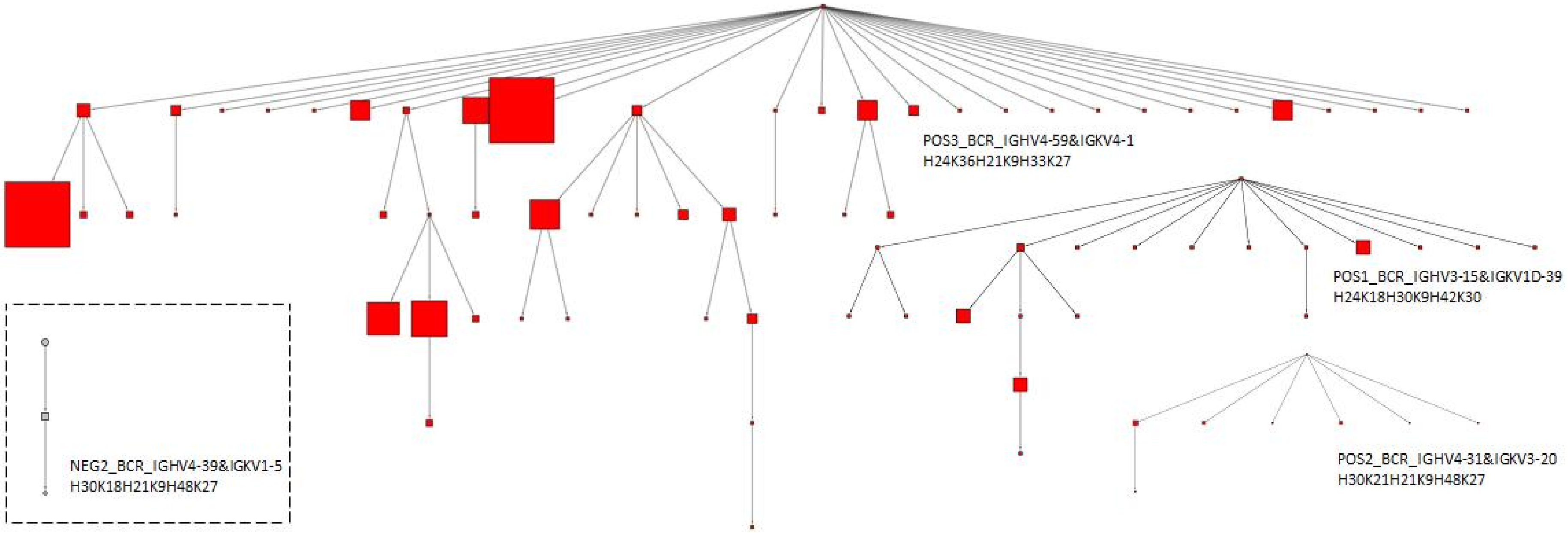

Supplementary Figure 3. Affinity maturation in positive (red) versus negative (grey) control B-cells. The three lineage trees in red are representative lineages for the three positive control samples (one tree per sample). Only one negative control sample (NEG2_BCR) has a lineage tree with depth≥2, shown as the grey tree inside the dashed box. Each lineage tree is named by sample name followed by V_H gene and V_L gene name and finally the length of each CDR with the order: CDRH1, CDRL1, CDRH2, CDRL2, CDRH3, CDRL3. Annotations (e.g., node name, node size, SHM, etc.) are omitted due to limited space.

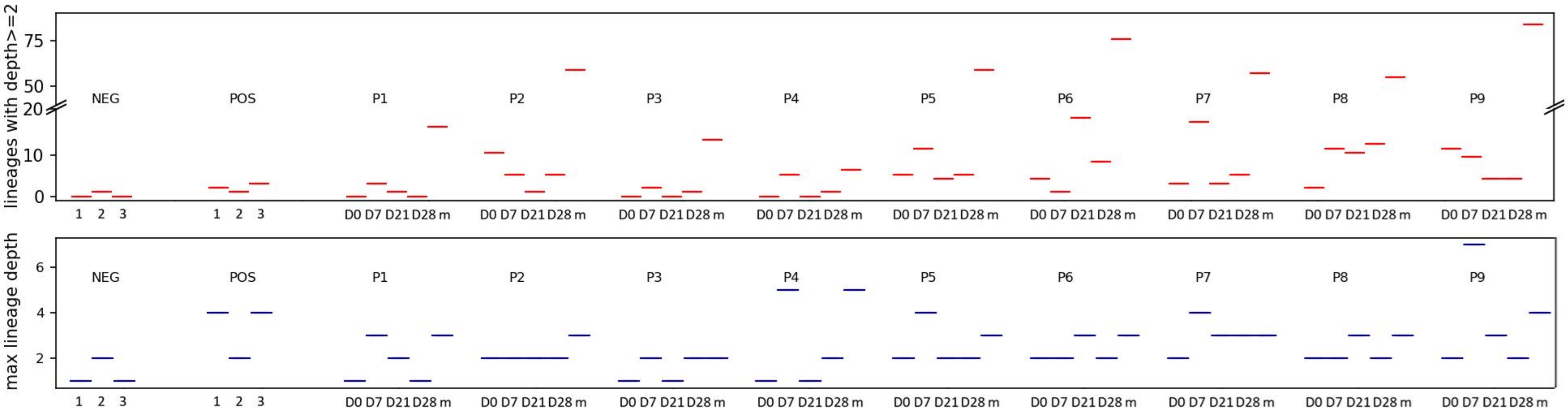

Supplementary Figure 4. Plots of lineage statistics of the B-cell repertoires from each vaccinated individual (P1-P9) at each time-point (D0, D7, D21, D28, merged), as well as three negative (NEG1-3) and three positive (POS1-3) controls.

a

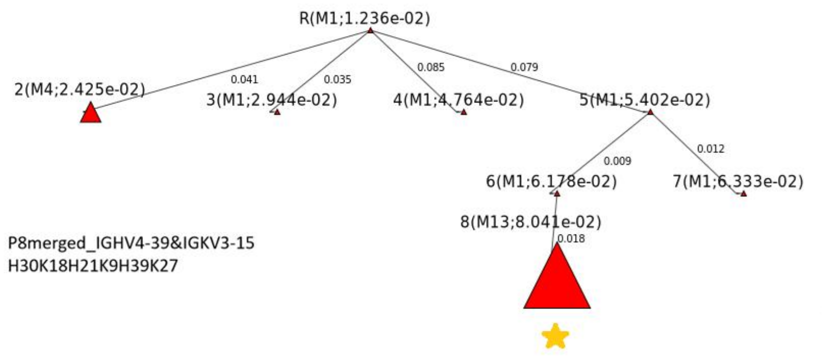

b

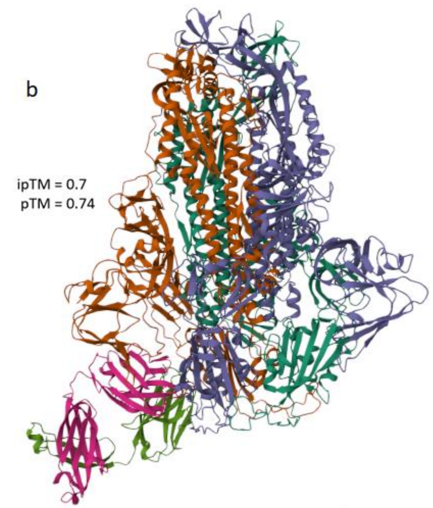

c

Entry	Chain	RMSD	TM-score	Identity	Aligned Residues	Sequence Length	Modeled Residues
P8node8	A	-	-	-	-	120	120
RBD-11	A	0.44	0.99	77%	120	127	127
RBD-9	A	0.5	0.99	73%	120	121	121
S1n-3	A	0.55	0.98	74%	119	125	125
S1n-2	A	0.55	0.98	71%	119	124	124
S2-3	A	0.6	0.98	70%	120	124	124
S2-2	A	0.55	0.97	70%	118	119	119

d

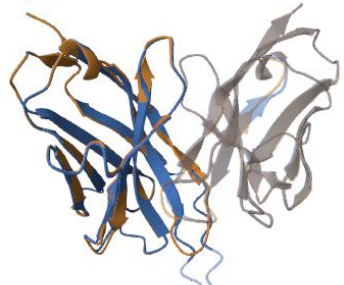

e

Entry	Chain	RMSD	TM-score	Identity	Aligned Residues	Sequence Length	Modeled Residues
P8node8	B	-	-	-	-	107	107
RBD-11	B	0.29	0.99	77%	106	106	106
RBD-2	B	0.27	0.99	85%	106	106	106
RBD-12	B	0.28	1	84%	107	107	107
RBD-15	B	0.27	1	85%	107	108	108
S2-3	B	0.28	0.99	71%	107	108	108

f

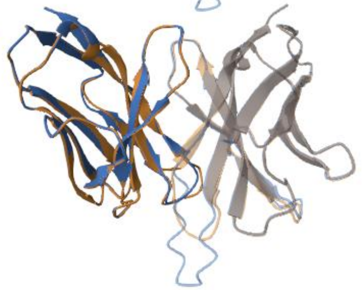

Supplementary Figure 5. An example of identified candidate with high structural similarity to verified high-affinity antibody targeting RBD.
a) The large, deep and expanded lineage tree from P8 where the candidate (node 8, highlighted with star) was selected. **b)** AlphaFold3 prediction of the interaction between the candidate and the SARS-CoV2 Spike protein trimer, with predicted binding-site at the RBD of the S1 subunit. **c)** Pairwise structural alignment results of the candidate and the top matches on heavy chain (chain A). **d)** Visualization of the alignment between the candidate and the RBD-11 antibody on heavy chain. **e)** Pairwise structural alignment results of the candidate and the top matches on light chain (chain B). **f)** Visualization of the alignment between the candidate and the RBD-11 antibody on light chain.

Supplementary Tables

Supplementary Table 1. Pairwise 3D structural alignment results of the selected candidate (P4node20) with 24 S-binding high-affinity antibodies which have been experimentally verified. **The top match antibody is highlighted in red.**

Entry	Chain	RMSD	TM-score	Identity	Aligned Residues	Sequence Length	Modeled Residues	Chain	RMSD	TM-score	Identity	Aligned Residues	Sequence Length	Modeled Residues
P4node20	A (H)	-	-	-	-	119	119	B (L)	-	-	-	-	107	107
S2-5	A (H)	1.14	0.93	48%	118	123	123	B (L)	0.58	0.98	60%	107	113	113
S2-4	A (H)	1.01	0.94	47%	118	120	120	B (L)	0.43	0.99	53%	107	112	112
S2-3	A (H)	0.45	0.99	71%	119	124	124	B (L)	0.33	0.99	60%	107	108	108
S2-2	A (H)	0.13	1	96%	119	119	119	B (L)	0.1	1	94%	107	107	107
S2-1	A (H)	1.38	0.91	47%	116	121	121	B (L)	1.78	0.89	43%	103	115	115
S1n-3	A (H)	0.39	0.99	77%	119	125	125	B (L)	1.32	0.92	45%	106	110	110
S1n-2	A (H)	0.3	1	83%	119	124	124	B (L)	1.36	0.91	44%	106	110	110
S1n-1	A (H)	1.1	0.93	51%	118	119	119	B (L)	1.42	0.91	46%	105	110	110
RBD-16	A (H)	1.13	0.93	43%	118	127	127	B (L)	1.38	0.91	43%	106	111	111
RBD-15	A (H)	1.08	0.93	52%	118	121	121	B (L)	0.35	0.99	64%	107	108	108
RBD-14	A (H)	1.11	0.91	51%	115	117	117	B (L)	1.4	0.91	40%	105	108	108
RBD-13	A (H)	1.07	0.93	49%	118	128	128	B (L)	0.28	1	80%	107	108	108
RBD-12	A (H)	0.99	0.95	48%	117	122	122	B (L)	0.39	0.99	62%	107	107	107
RBD-11	A (H)	0.38	0.99	78%	119	127	127	B (L)	0.35	0.98	68%	106	106	106
RBD-10	A (H)	1.15	0.92	50%	117	117	117	B (L)	0.33	0.99	73%	107	108	108
RBD-9	A (H)	0.34	0.99	75%	119	121	121	B (L)	0.18	1	79%	107	107	107
RBD-8	A (H)	1.11	0.93	50%	118	127	127	B (L)	0.19	1	77%	107	107	107
RBD-7	A (H)	0.34	0.99	77%	119	124	124	B (L)	1.44	0.91	46%	104	111	111
RBD-6	A (H)	1.19	0.93	50%	118	127	127	B (L)	0.17	1	78%	107	107	107
RBD-5	A (H)	1.34	0.92	48%	116	121	121	B (L)	1.4	0.91	40%	105	108	108
RBD-4	A (H)	1.05	0.94	44%	117	127	127	B (L)	0.22	1	77%	107	107	107
RBD-3	A (H)	0.58	0.98	79%	118	124	124	B (L)	1.41	0.91	48%	104	112	112
RBD-2	A (H)	1.12	0.94	49%	116	121	121	B (L)	0.33	0.98	64%	106	106	106
RBD-1	A (H)	1	0.95	48%	117	127	127	B (L)	1.41	0.91	48%	104	111	111

Supplementary Table 2. Pairwise 3D structural alignment results of the selected candidate (P8node8) with 24 S-binding high-affinity antibodies which have been experimentally verified. **The top match antibody is highlighted in red.** Since both chains should be considered, the top match is not necessarily the closest match in a single chain but must be the closest overall, at least with RMSD < 0.5 in both chains.

Entry	Chain	RMSD	TM-score	Identity	Aligned Residues	Sequence Length	Modeled Residues	Chain	RMSD	TM-score	Identity	Aligned Residues	Sequence Length	Modeled Residues
P8node8	A (H)	-	-	-	-	120	120	B (L)	-	-	-	-	107	107
S2-5	A (H)	1.28	0.91	44%	117	123	123	B (L)	0.6	0.98	64%	107	113	113
S2-4	A (H)	1.01	0.94	50%	118	120	120	B (L)	0.42	0.99	51%	107	112	112
S2-3	A (H)	0.6	0.98	70%	120	124	124	B (L)	0.28	0.99	71%	107	108	108
S2-2	A (H)	0.55	0.97	70%	118	119	119	B (L)	0.3	0.99	65%	107	107	107
S2-1	A (H)	1.5	0.9	44%	115	121	121	B (L)	1.74	0.89	45%	103	115	115
S1n-3	A (H)	0.55	0.98	74%	119	125	125	B (L)	1.31	0.92	43%	106	110	110
S1n-2	A (H)	0.55	0.98	71%	119	124	124	B (L)	1.34	0.91	43%	106	110	110
S1n-1	A (H)	1.12	0.92	50%	117	119	119	B (L)	1.4	0.91	43%	105	110	110
RBD-16	A (H)	1.12	0.92	41%	118	127	127	B (L)	1.35	0.91	43%	106	111	111
RBD-15	A (H)	1.03	0.93	50%	118	121	121	B (L)	0.27	1	85%	107	108	108
RBD-14	A (H)	1.05	0.91	50%	115	117	117	B (L)	1.39	0.91	44%	105	108	108
RBD-13	A (H)	1.2	0.92	47%	117	128	128	B (L)	0.34	0.99	67%	107	108	108
RBD-12	A (H)	1.08	0.94	42%	117	122	122	B (L)	0.28	1	84%	107	107	107
RBD-11	A (H)	0.44	0.99	77%	120	127	127	B (L)	0.29	0.99	77%	106	106	106
RBD-10	A (H)	1.22	0.91	47%	114	117	117	B (L)	0.39	0.99	61%	107	108	108
RBD-9	A (H)	0.5	0.99	73%	120	121	121	B (L)	0.3	0.99	65%	107	107	107
RBD-8	A (H)	1.13	0.92	52%	118	127	127	B (L)	0.3	0.99	65%	107	107	107
RBD-7	A (H)	0.63	0.98	78%	120	124	124	B (L)	1.44	0.91	45%	104	111	111
RBD-6	A (H)	1.15	0.92	51%	117	127	127	B (L)	0.3	0.99	65%	107	107	107
RBD-5	A (H)	1.18	0.92	48%	118	121	121	B (L)	1.39	0.91	46%	105	108	108
RBD-4	A (H)	1.17	0.93	38%	117	127	127	B (L)	0.3	0.99	63%	107	107	107
RBD-3	A (H)	0.74	0.96	78%	118	124	124	B (L)	1.41	0.91	47%	104	112	112
RBD-2	A (H)	1.02	0.94	45%	116	121	121	B (L)	0.27	0.99	85%	106	106	106
RBD-1	A (H)	1.03	0.94	43%	117	127	127	B (L)	1.41	0.91	46%	104	111	111
